# Comparative mitogenomics and phylogenetic implications of *Dawkinsia apsara* and *Dawkinsia denisonii* (Cypriniformes, Cyprinidae)

**DOI:** 10.3897/zookeys.1270.182564

**Published:** 2026-02-18

**Authors:** Xiao Ma, Yang Xu, Xiao-Die Chen, Cheng-He Sun, Chang-Hu Lu

**Affiliations:** 1 College of Life Sciences, Nanjing Forestry University, Nanjing 210037, China College of Life Sciences, Nanjing Forestry University Nanjing China

**Keywords:** Comparative genomics, control-region variation, *
Dawkinsia
*, phylogeny, mito­chondrial genome

## Abstract

The classification of the *Puntius*-*Dawkinsia* complex has long been controversial due to overlapping morphological characteristics and inconsistent phylogenetic tree topology based on short mtDNA fragments. In order to better clarify the species relationship within the genus *Dawkinsia*, the complete mitochondrial genome of *Dawkinsia apsara* was determined for the first time, and the mitochondrial genome of *Dawkinsia denisonii* was newly sequenced independently for comparative analysis. The results showed that the nucleotide composition of mtDNA of *D. apsara* (16,470 bp) and *D. denisonii* (16,900 bp) was similar. The total AT content of the two fish species was 58.9% and 58.2%, respectively. RSCU and amino acid composition showed that A/T-ending synonymous codons, and Leu was the most abundant amino acid. However, there are significant differences in the length and structure of the control region between the two species, especially in the tandem repeats and conserved structural units. The phylogenetic tree constructed based on ML and BI methods strongly supported the monophyly of genus *Dawkinsia*. The *Dawkinsia denisonii* was clustered with *D. chalakkudiensis* within the genus, followed by *D. tambraparniei* and *D. apsara*, and obtained a higher phylogenetic resolution than previous studies between *Dawkinsia* and its related genera such as *Pethia* and *Puntius*. This study provides the first complete mitochondrial genome of *D. apsara*, reveals the structural variations in the genome, particularly within the control regions, among the closely related species of *Dawkinsia*, enriches the phylogenetic data of the genus *Dawkinsia*, and provides new mitochondrial genome-scale evidence for the long-controversial *Puntius*-*Dawkinsia* complex.

## Introduction

Species identification is fundamentally based on morphological evidence and is increasingly supported by molecular biological approaches within an integrative taxonomic framework. Among them, the mitochondrial genome has become an ideal molecular marker for phylogenetic research and species identification due to its maternal inheritance, compact structure, and moderate evolutionary rate (Sun et al. 2024; Liu et al. 2025). The mitochondrial DNA of fish is usually a double-stranded circular molecule of approximately 15–20 kb (Boore 1999), containing 13 protein-coding genes (PCGs), two rRNA genes, 22 tRNA genes and a non-coding control region (D-loop) (Pereira 2000). Although gene fragments such as *COXI* (Chang et al. 2017; Sheraliev and Peng 2021) and 12S rRNA (Miya et al. 2015) are often used as DNA barcodes, the complete mitochondrial genome can provide more comprehensive and uniform genetic variation information, effectively overcoming the phylogenetic uncertainty of single gene markers (Chen et al. 2021; Xie et al. 2022). In recent years, thanks to the popularity of high-throughput sequencing and assembly technology, the number of fish mitochondrial genome data has grown rapidly (Zhang et al. 2023). Osteichthyes has accumulated a large number of mitochondrial genomes and covered dozens of order-level groups, providing sufficient data for cross-group phylogenetic and molecular identification studies (Wang et al. 2022; Zhu et al. 2024).

Cyprinidae is one of the most diverse groups in freshwater fish, and the phylogenetic relationship within it, especially the *Puntius*-*Dawkinsia* complex, has long been controversial (Ren et al. 2020; Sudasinghe et al. 2023). Since the work of Pethiyagoda et al. (2012), who analyzed 16S rRNA and *Cytb* gene fragments from 31 South Asian species traditionally referred to *Puntius*, it has become evident that the conventional concept of *Puntius* represents a highly polyphyletic assemblage. Based on molecular evidence and a combination of stable morphological characters—including the absence of rostral barbels, a smooth last unbranched dorsal-fin ray, and a complete lateral line bearing 18–22 lateral line scales—Pethiyagoda and colleagues established *Dawkinsia* as a distinct genus. Later, Tan and Armbruster (2018) placed *Dawkinsia* within the subfamily Smiliogastrinae under a broader phylogenetic framework, further clarifying its evolutionary affinities with *Pethia*, *Puntius*, *Puntigrus*, and related genera. However, the genomic divergence among many species within *Dawkinsia* has not yet been systematically evaluated, and their species boundaries as well as potential molecular diagnostic characters remain unclear. Previous studies have largely relied on short mitochondrial fragments or a limited number of nuclear markers, lacking comprehensive examinations of control-region variation, genome-wide structural differences, and the phylogenetic resolving power of complete mitogenomes (Sudasinghe et al. 2021). As a result, phylogenetic reconstructions have often yielded unstable node support or conflicting topologies across studies, leaving the evolutionary relationships within the complex unresolved.

The *Dawkinsia apsara* Katwate, Knight, Anoop, Rajeev Raghavan & Dahanukar, 2020 is distinguished by its metallic purple sheen and black lateral spots, whereas *D. denisonii* (Day, 1865) is well known for the longitudinal scarlet stripes extending across the head and tail (Raghavan et al. 2013; Ali et al. 2015; Katwate et al. 2020). Although the complete mitochondrial genome of *D. denisonii* has been preserved in GenBank (AP011244), the species is widely traded in the ornamental fish market and has been reported to have hidden lineages (Collins et al. 2012; Jain et al. 2022). In order to verify the stability of the existing mitochondrial genome and evaluate potential intraspecific divergence, we compared the existing sequences with the new test results. Therefore, in this study, we sequenced the complete mitochondrial genome of *D. apsara* for the first time and independently re-sequenced that of *D. denisonii* to verify the reliability of existing data. We compared the two species in terms of genome structure, protein-coding gene evolutionary patterns, and D-loop organization, and further reconstructed genus-level phylogenetic relationships using available mitogenome data from the subfamily Smiliogastrinae. Complete mitochondrial genomes are expected to reveal species-level differences that cannot be detected by short fragments and to provide new taxonomic and evolutionary insights into the systematic placement of *Dawkinsia* and its close relatives.

## Materials and methods

### Sample collection and DNA extraction

Living samples of *D. apsara* and *D. denisonii* were obtained from the Fangcun Flower, Bird, Fish and Insect Market (Guangzhou), and voucher specimens were deposited at the College of Life Sciences, Nanjing Forestry University (Nanjing, China) under accession numbers NFU-Daw-1 and NFU-Daw-2, respectively. In view of the common mislabeling and hybridization problems in the ornamental fish trade, we compared the key characteristics such as body color, body side markings, presence and length of dorsal fin filaments, and body shape ratio of the samples. Among them, *D. apsara* has the characteristics of metallic purple luster and black spots on the body side, while *D. denisonii* takes the bright red longitudinal striations throughout the head to the caudal peduncle as the typical morphological markers (Ali et al. 2015; Katwate et al. 2020). The muscle tissue samples of each species were preserved in anhydrous ethanol, and high-quality total DNA was extracted from the tissues using the CTAB method (Porebski et al. 1997).

### Mitochondrial genome sequencing and assembly

DNA samples meeting the quality standards were sent to Personal Biotechnology Co., Ltd.​ (Shanghai, China) for 350 bp small fragment library construction and high-throughput sequencing. PE150 sequencing was performed based on the Illumina NovaSeq 6000 platform, and the sequencing data was retained by quality control to retain valid reads greater than 6–10 GB. The raw reads were subjected to strict quality control using Fastp v0.23.0 software, and the ‘-careful’ mode of SPAdes v3.15.0 software (Bankevich et al. 2012; Nurk et al. 2017) was used to de novo assemble all clean data to obtain complete and circular mitochondrial genome sequences.

### Genome annotation and data submission

The assembled circular mitochondrial genome was submitted to the MITOS2 online server (http://mitos2.bioinf.uni-leipzig.de) for automated gene annotation, and the genetic code was set as the vertebrate mitochondrial genetic code table (Bernt et al. 2013). Subsequently, the annotation results were compared with the NCBI NR database by BLAST to verify the accuracy, and the secondary structure of tRNA gene was further verified by tRNAscan-SE software (Lowe and Eddy 1997). Finally, the mitochondrial genome map was drawn using the MitoFish online website (https://mitofish.aori.u-tokyo.ac.jp/) (Zhu et al. 2023). The complete mitochondrial genome sequences of the assembled and annotated two species of *Dawkinsia* were submitted to the NCBI GenBank database.

### Genome analysis

PhyloSuite v1.2.1 was used to calculate the length of each protein-coding gene, rRNA gene, tRNA gene and D-loop region, base composition (A, T, G, C content and AT content, GC content, etc.) and relative synonymous codon usage (RSCU), and identify the start codon and stop codon of all protein-coding genes (Zhang et al. 2020). MEGA v11 was used to calculate the relative usage of synonymous codons of each protein-coding gene in the two species, and the number of effective codons was counted to evaluate the codon usage preference (Kumar et al. 1994). In order to explore the evolutionary dynamics of protein-coding genes, DnaSP v5 was used to calculate the ratio of non-synonymous substitution rate to synonymous substitution rate of each protein-coding gene, and the selection pressure of the gene was evaluated (Librado and Rozas 2009).

### Phylogenetic analysis

The mitochondrial genome sequences of Smiliogastrinae fishes were downloaded from NCBI GenBank database, and *Schizothorax biddulphi* and *S. oconnori* in another subfamily Schizothoracinae of the same family were selected as outgroups (Table 1). The MAFFT tool was used for multiple sequence alignment (Katoh and Standley 2013). The PCG data set was further optimized using MACSE (Ranwez et al. 2018), and the unclear comparison regions were trimmed with Gblocks (Talavera and Castresana 2007). At the same time, trimAl was used to trim the RNA genes sequence, and Gblocks was used to trim the exogenous sequence. After concatenating the data, the ModelFinder (Kalyaanamoorthy et al. 2017) in PhyloSutie v1.2.1 was used to select the optimal partitioning strategy and model, and the IQ-TREE v1.6.8 (Nguyen et al. 2015) was used to construct the maximum likelihood (ML) phylogenetic tree. The support rate of each branch was evaluated by 50000 ultra-fast bootstrap tests, and the bootstrap value of each branch was calculated. The Bayesian inference (BI) phylogenetic tree was constructed using MrBayes v3.2.6 (Ronquist et al. 2012), and Markov chain Monte Carlo simulation was performed. Two independent Markov chains were set to run a total of 10,000,000 generations. Sampling was performed once every 1,000 generations. The first 25% of the samples were discarded as aging samples. The posterior probability was calculated according to the remaining samples, and the Bayesian posterior probability value of each node was calculated. Finally, the iTOL (https://itol.embl.de/) online website was used to beautify the phylogenetic tree (Letunic and Bork 2016).

**Table 1.** Mitochondrial genome information for the 37 Smiliogastrinae species and 2 outgroup species involved.

Subfamily	Species	Size (bp)	ID
Smiliogastrinae	*Barbodes aurotaeniatus*	16562	NC_031619
	*Barbodes binotatus*	16573	NC_034755
	*Barbodes semifasciolatus*	16594	NC_020096
	*Barboides gracilis*	16566	NC_031550
	*Clypeobarbus pleuropholis*	16570	NC_031627
	*Dawkinsia apsara*	16470	PV684861
	*Dawkinsia chalakkudiensis*	16989	NC_018566
	*Dawkinsia denisonii*	16900	PV904161
	*Dawkinsia tambraparniei*	16610	NC_031614
	*Enteromius callipterus*	16859	AP009313
	*Enteromius eburneensis*	16678	NC_031617
	*Enteromius fasciolatus*	16566	NC_031616
	*Enteromius guirali*	16793	AP009314
	*Enteromius hulstaerti*	16775	NC_031530
	*Enteromius pobeguini*	16933	NC_033914
	*Enteromius thysi*	16688	NC_069976
	*Enteromius trimaculatus*	16417	NC_008666
	*Hampala macrolepidota*	16766	NC_029149
	*Hampala salweenensis*	16913	NC_061683
	*Oliotius oligolepis*	16636	NC_066909
	*Oreichthys crenuchoides*	16894	NC_033915
	*Osteobrama cotio*	16584	AP011260
	*Osteobrama cunma*	16650	NC_031559
	*Osteobrama feae*	16578	NC_031560
	*Pethia conchonius*	17082	NC_022856
	*Pethia padamya*	16792	NC_066910
	*Pethia stoliczkana*	16996	NC_072568
	*Pethia ticto*	17302	NC_008658
	*Puntigrus partipentazona*	16597	NC_031610
	*Puntigrus tetrazona*	16550	NC_010110
	*Puntius eugrammus*	16847	NC_031611
	*Puntius paucimaculatus*	16590	OR264466
	*Puntius sachsii*	16587	MZ364158
	*Puntius sahyadriensis*	16798	NC_033916
	*Puntius snyderi*	16578	NC_020097
	*Systomus orphoides*	16593	NC_031527
	*Systomus sarana*	16590	KU886061
Schizothoracinae	*Schizothorax biddulphi*	16585	NC_017873
	*Schizothorax oconnori*	16590	NC_020781

## Results

### Mitochondrial genome structure

**Figure 1.**
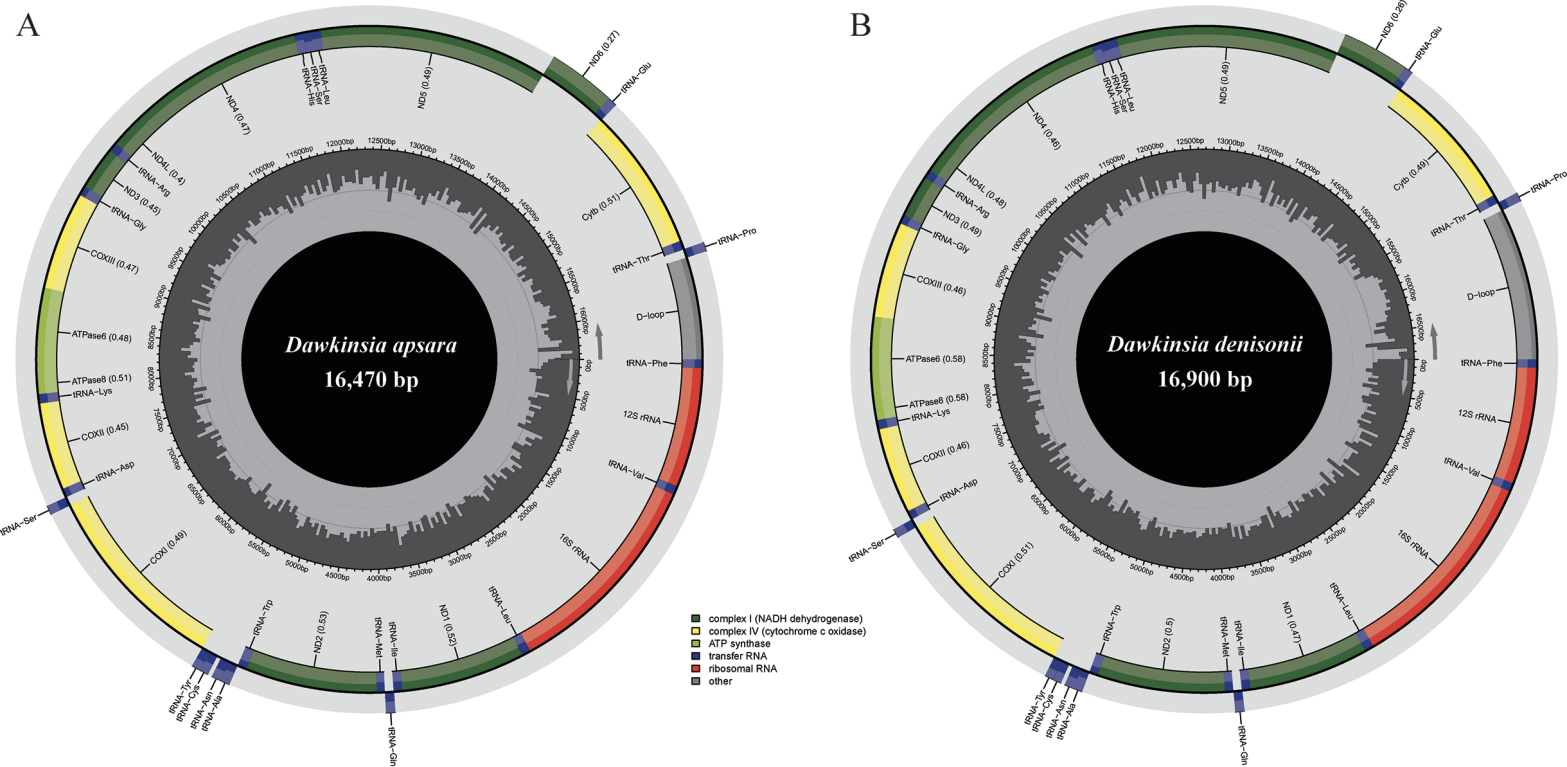
Mitochondrial genome information diagram of *D. apsara* (**A**) and *D. denisonii* (**B**). Dark green blocks: complex I (NADH dehydrogenase), yellow blocks: complex IV (cytochrome c oxidase), light green blocks: ATP synthase, blue blocks: transfer RNA, red blocks: ribosomal RNA, gray blocks: other.

The complete mitochondrial genomes of *D. apsara* and *D. denisonii* showed a typical circular double-stranded structure, including 37 genes (13 PCGs, 22 tRNA genes and two rRNA genes) and a major non-coding control region (Fig. 1). The genome length of *D. apsara* is 16,470 bp, and the D-loop region is 814 bp. The genome length of *D. denisonii* is 16,900 bp, and the D-loop region is 1,246 bp. Most of the genes were located in the heavy chain, and only *ND6* and eight tRNA were distributed in the light chain (Table 2). In addition, there are many gene intervals and overlaps in the genome, among which there are seven base overlap regions between *ATP8* and *ATP6* and between *ND4L* and *ND4*. The largest intergenic spacer was located between *tRNA-Cys* and *tRNA-Tyr*, with lengths of 31 bp and 32 bp, respectively.

**Table 2.** Mitochondrial genome characteristics of *D. apsara* and *D. denisonii*.

Gene	Position		Size	Intergenic nucleotides	Codon		Anticodon	Strand
	From	To			Start	Stop		
*tRNA-Phe*	1\1	69\69	69\69	0\0			GCT	H
*12S rRNA*	70\70	1024\1026	955\957	0\0				H
*tRNA-Val*	1025\1027	1096\1098	72\72	0\0			CAG\CGG	H
*16S rRNA*	1097\1099	2793\2790	1697\1692	0\0				H
*tRNA-Leu*	2794\2791	2867\2866	74\76	0\0			ACT	H
*ND1*	2869\2868	3843\3842	975\975	1\1	ATG	TAA		H
*tRNA-Ile*	3855\3847	3925\3918	71\72	11\4			GGA	H
*tRNA-Gln*	3924\3917	3994\3987	71\71	-2\-2			TAG	L
*tRNA-Met*	3996\3989	4059\4057	64\69	1\1			GGC	H
*ND2*	4065\4058	5109\5099	1045\1042	5\0	ATG	T		H
*tRNA-Trp*	5110\5100	5182\5170	73\71	0\0			GTG\AGG	H
*tRNA-Ala*	5184\5172	5252\5240	69\69	1\1			GAG\AGG	L
*tRNA-Asn*	5254\5242	5326\5314	73\73	1\1			TAG	L
*tRNA-Cys*	5359\5346	5425\5411	67\66	32\31			AAC\AGC	L
*tRNA-Tyr*	5425\5411	5493\5479	69\69	-1\-1			GGT	L
*COXI*	5495\5481	7045\7031	1551\1551	1\1	GTG	TAA		H
*tRNA-Ser*	7046\7032	7116\7102	71\71	0\0			AAG	L
*tRNA-Asp*	7118\7104	7189\7175	72\72	1\1			AAG	H
*COXII*	7195\7189	7885\7879	691\691	5\13	ATG	T		H
*tRNA-Lys*	7886\7880	7961\7955	76\76	0\0			CTC\CAC	H
*ATP8*	7964\7957	8128\8121	165\165	2\1	ATG	TAG		H
*ATP6*	8122\8115	8804\8797	683\683	-7\-7	ATG	TA		H
*COXIII*	8805\8798	9589\9582	785\785	0\0	ATG	TA		H
*tRNA-Gly*	9590\9583	9663\9655	74\73	0\0			ATC	H
*ND3*	9664\9656	10012\10004	349\349	0\0	ATA	T		H
*tRNA-Arg*	10013\10005	10083\10074	71\70	0\0			AGG\	H
*ND4L*	10084\10075	10380\10371	297\297	0\0	ATG	TAA		H
*ND4*	10374\10365	11754\11745	1381\1381	-7\-7	ATG	T		H
*tRNA-His*	11755\11746	11823\11814	69\69	0\0			GTA	H
*tRNA-Ser*	11824\11815	11891\11883	68\69	0\0			GGG\AAG	H
*tRNA-Leu*	11893\11885	11965\11957	73\73	1\1			GCT	H
*ND5*	11967\11960	13784\13780	1818\1821	1\2	ATG	TAA		H
*ND6*	13781\13777	14302\14298	522\522	-4\-4	GTG	TAG		L
*tRNA-Glu*	14303\14299	14371\14366	69\68	0\0			ATT\GTT	L
*Cytb*	14377\14373	15513\15509	1137\1137	5\6	ATG	TAA		H
*tRNA-Thr*	15517\15514	15588\15584	72\71	3\4			GCC	H
*tRNA-Pro*	15587\15584	15656\15654	70\71	-2\-1			CAG	L
D-loop	15657\15655	16470\16900	814\1246	0\0				H

Comparison between the newly sequenced mitogenome and the published *D. denisonii* reference (AP011244) revealed substantial mitochondrial variation. Across the 16,915 bp alignment, 881 polymorphic sites (SNPs) and 22 independent InDel events were detected, corresponding to 881 nucleotide differences and a genome-wide p-distance of 0.0524. These values indicate a level of divergence far exceeding typical sequencing noise and instead suggest marked intraspecific mitochondrial differentiation.

### Difference of D-loop

Marked structural divergence was observed in the mitochondrial D-loop between *D. apsara* and *D. denisonii*. The D-loop of *D. apsara* was relatively short (814 bp), whereas that of *D. denisonii* was substantially longer (1246 bp). This pronounced length difference was primarily attributable to the expansion of a tandem repeat array in *D. denisonii*. A conserved repeat unit of approximately 60 bp (ACATAATGTATTAGTACATATATGTATTATCACCATAAATTTTATTTAGACCATAAAGCAGGTACTAAATA TTAAGAT) was identified and duplicated six times in the control region of *D. denisonii*, while this repeat motif was absent in *D. apsara*. Apart from the repeat array, the remaining regions of the control region showed no large-scale structural rearrangements.

### Mitochondrial genome base preference

**Figure 2.**
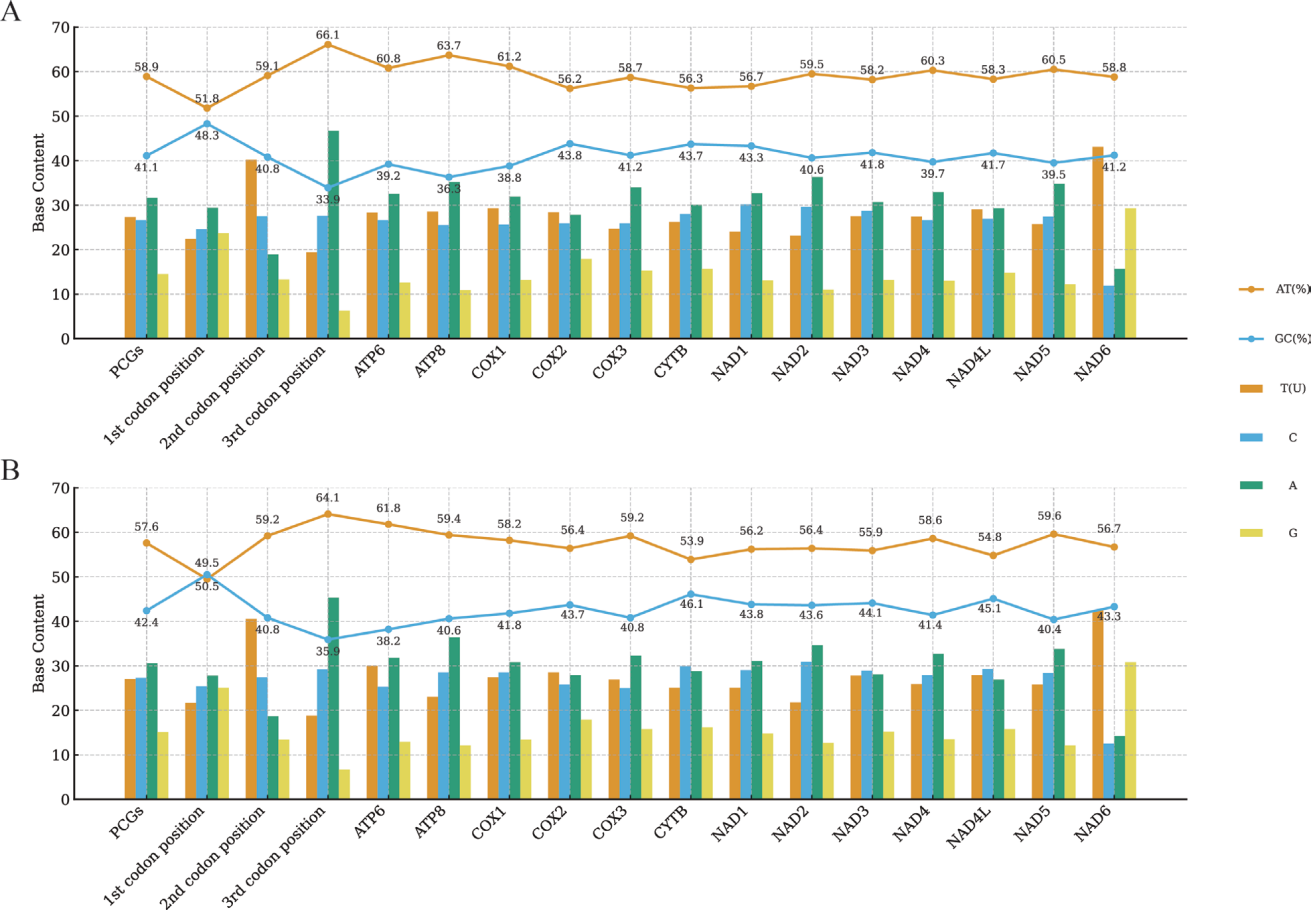
The base composition and quantities, as well as AT content and CG content, of the 13 PCGs in *D. apsara* (**A**) and *D. denisonii* (**B**). X axis: different genomic regions/codon positions. Y axis: percentage of base content (%).

The genome-wide AT content of *D. apsara* was slightly higher, 58.9%, and the GC content was 41.1%; the genome-wide AT content of *D. denisonii* was 58.2%, and the GC content was 41.8% (Table 3). In both species, protein-coding genes, rRNA, tRNA and D-loop regions showed similar AT preference, and the AT content in D-loop region was the highest, reaching 70.1% and 73.4% in *D. apsara* and *D. denisonii*, respectively (Table 3). Fig. 2 shows the base composition and quantities, as well as AT content and CG content, of the 13 PCGs in *D. apsara* and *D. denisonii*. The analysis of base skew showed that the two species showed consistent positive AT skew and negative GC skew in the whole genome and most regions. The skew values of AT were all positive (*D. apsara* 0.138, *D. denisonii* 0.134), indicating that the content of adenine is higher than that of thymine; the GC skewness values were all negative (*D. apsara* -0.273, *D. denisonii* -0.266), indicating that the content of cytosine is higher than that of guanine.

**Table 3.** Nucleotide composition of *D. apsara* and *D. denisonii* mitochondrial genome.

Species	Regions	Size (bp)	T(U)/%	C/%	A/%	G/%	AT/%	GC/%	AT skew	GC skew
*D. apsara*	Full genome	16470	25.4	26.2	33.5	14.9	58.9	41.1	0.138	-0.273
	PCGs	11391	27.3	26.6	31.6	14.5	58.9	41.1	0.073	-0.296
	rRNAs	2652	19.9	24.4	36.1	19.6	56	44	0.288	-0.108
	tRNAs	1557	27.2	20.6	29.3	22.9	56.5	43.5	0.038	0.053
	D-loop	814	34.4	18.8	35.7	11.1	70.1	29.9	0.019	-0.258
*D. denisonii*	Full genome	16900	25.2	26.5	33	15.3	58.2	41.8	0.134	-0.266
	PCGs	11391	27	27.3	30.6	15.1	57.6	42.4	0.063	-0.289
	rRNAs	2649	19.3	25.1	35.4	20.2	54.7	45.3	0.295	-0.107
	tRNAs	1560	27	21.2	29.1	22.7	56.1	43.9	0.038	0.034
	D-loop	1246	34.6	15.5	38.8	11.2	73.4	26.6	0.057	-0.161

### Mitochondrial genome codon usage frequency

**Figure 3.**
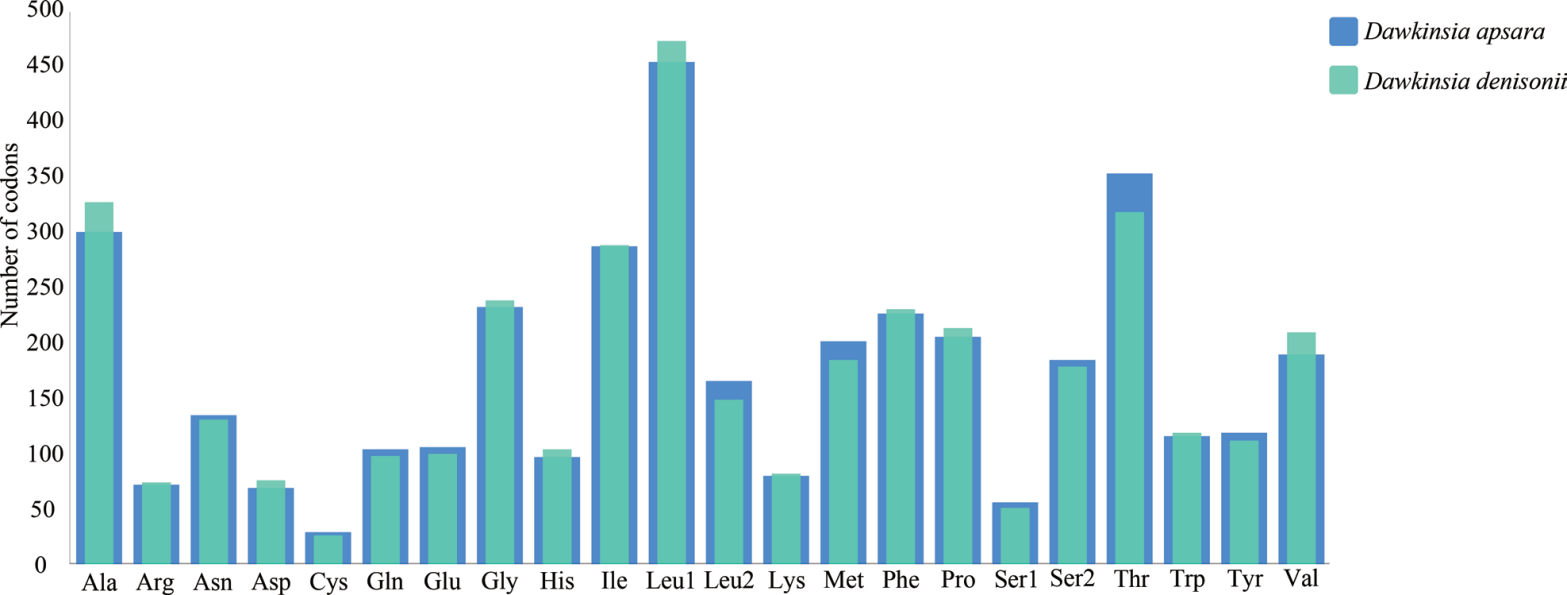
Codon distribution of *D. apsara* and *D. denisonii* mitogenome. Numbers on the Y-axis refer to the total number of codons, and codon families are provided on the X-axis.

Codon usage frequency analysis showed that the most frequently used amino acids in both species were leucine, followed by isoleucine, threonine, and alanine (Fig. 3). In the analysis of the relative usage of synonymous codons, both species were significantly inclined to use codons ending with A or U (Table 4). For example, the RSCU values of CUA encoding leucine, GUA encoding valine, CCA encoding proline, GGA encoding glycine, and CGA encoding arginine were all greater than 2, showing a strong preference for usage. At the same time, synonymous codons ending with G or C are mostly less used, and the RSCU value is generally less than 0.6. It is worth noting that although AUA usually encodes methionine, its use frequency (RSCU: 1.71 and 1.65) is much higher than the standard AUG methionine codon (RSCU: 0.29 and 0.35) in the mitochondrial genomes of these two fish species.

**Table 4.** Comparison of RSCU between *D. apsara* and *D. denisonii*.

Codon	RSCU		Codon	RSCU	
	*D. apsara*	*D. denisonii*		D. apsara	D. denisonii
UUU(F)	1.05	0.96	UAU(Y)	0.71	0.95
UUC(F)	0.95	1.04	UAC(Y)	1.29	1.05
UUA(L)	1.37	1.29	UAA(*)	2.86	2.86
UUG(L)	0.23	0.14	UAG(*)	1.14	1.14
CUU(L)	0.83	0.78	CAU(H)	0.54	0.42
CUC(L)	0.68	0.73	CAC(H)	1.46	1.58
CUA(L)	2.5	2.69	CAA(Q)	1.88	1.78
CUG(L)	0.39	0.37	CAG(Q)	0.12	0.22
AUU(I)	0.99	1.06	AAU(N)	0.62	0.56
AUC(I)	1.01	0.94	AAC(N)	1.38	1.44
AUA(M)	1.71	1.65	AAA(K)	1.82	1.93
AUG(M)	0.29	0.35	AAG(K)	0.17	0.07
GUU(V)	0.88	0.84	GAU(D)	0.64	0.55
GUC(V)	0.55	0.57	GAC(D)	1.36	1.45
GUA(V)	2.19	2.08	GAA(E)	1.7	1.7
GUG(V)	0.38	0.51	GAG(E)	0.3	0.3
UCU(S)	0.75	0.83	UGU(C)	0.55	0.38
UCC(S)	1.12	1.49	UGC(C)	1.45	1.62
UCA(S)	2.56	2.19	UGA(W)	1.71	1.71
UCG(S)	0.17	0.16	UGG(W)	0.29	0.29
CCU(P)	0.39	0.34	CGU(R)	0.56	0.38
CCC(P)	0.54	0.75	CGC(R)	0.39	0.54
CCA(P)	2.78	2.56	CGA(R)	2.89	2.92
CCG(P)	0.29	0.36	CGG(R)	0.17	0.16
ACU(T)	0.68	0.5	AGU(S)	0.3	0.29
ACC(T)	1.21	1.37	AGC(S)	1.1	1.04
ACA(T)	2.07	2.04	AGA(*)	0	0
ACG(T)	0.05	0.09	AGG(*)	0	0
GCU(A)	0.7	0.7	GGU(G)	0.36	0.35
GCC(A)	1.62	1.68	GGC(G)	0.62	0.69
GCA(A)	1.55	1.46	GGA(G)	2.27	2.18
GCG(A)	0.12	0.16	GGG(G)	0.76	0.79

### Protein-coding genes

**Figure 4.**
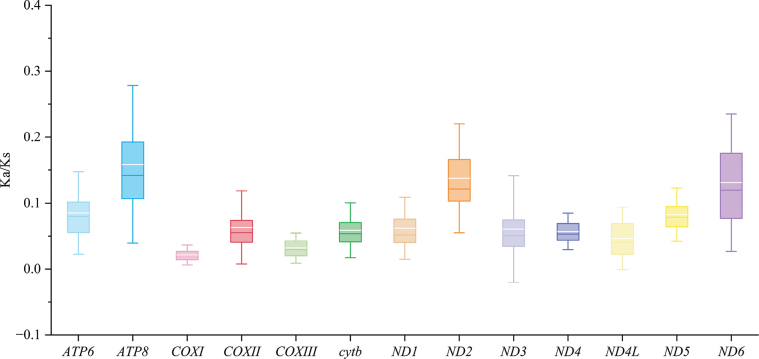
Analysis of Ka/Ks ratios for 13 protein-coding genes across 37 species of Smiliogastrinae. The white horizontal line is the mean line, and the horizontal line with the same color as the box is the median line.

It can be seen from Fig. 4 that Ka/Ks values of all PCGs are less than 1. Among them, *COXIII* showed the smallest Ka/Ks ratio (0.015), and *ND2* had the highest Ka/Ks ratio (0.070). *COXIII* and *ND2* represent the two extremes of molecular evolution: *COXIII* shows strong purification selection, while *ND2* shows relatively high evolutionary flexibility. In terms of protein-coding genes, the gene composition and order of the two species are highly conserved. The termination codon analysis showed that *ND2*, *COXII*, *ND3* and *ND4* genes were terminated by a single T base in both species. *ATP6* and *COXIII* genes use TA; the remaining genes such as *ND1*, *ND4L*, *ND5* and *Cytb* use the complete TAA or TAG termination codon (Table 2).

### Phylogenetic relationship

**Figure 5.**
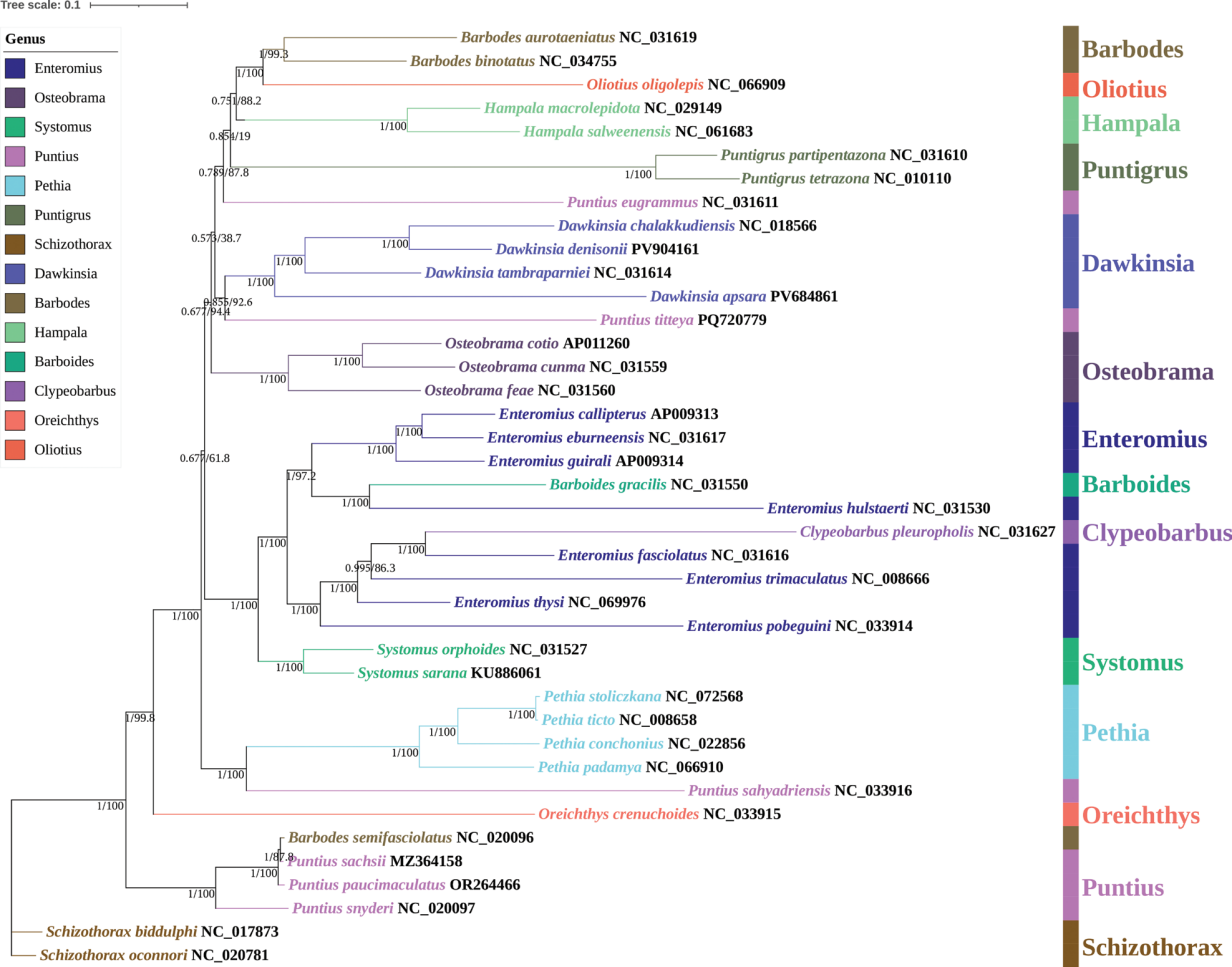
Phylogenetic tree inferred from Bayesian inference (BI) and maximum likelihood (ML) analyses based on the nucleotide sequences from 37 Smiliogastrinae fishes and two outgroups. Nodal support values are shown as BI posterior probabilities/ML bootstrap values.

Phylogenetic analysis clearly showed that all *Dawkinsia* species clustered into a monophyletic group with high support, confirming the effectiveness of the genus (Fig. 5). *D. denisonii* formed a strongly supported sister relationship with *D. chalakkudiensis*, and this pair subsequently clustered with *D. tambraparniei*; this three-species clade was then grouped with *D. apsara*. All *Dawkinsia* species formed a well-supported monophyletic group. At a higher level of classification, the phylogenetic tree revealed that the genus *Dawkinsia* was closely related to some species of *Puntigrus* and *Puntius* (such as *P. eugrammus*), which together constituted a strong monophyletic branch. This clade formed a sister group relationship with *Barbodes*, *Systomus*, *Pethia*, and other genera.

## Discussion

### Genome structure characteristics

The mitochondrial genome lengths of *D. apsara* and *D. denisonii* obtained in this study fall within the typical size range reported for fish mitogenomes, 15–19 kb (Gong et al. 2025). The control region length heterogeneity between *D. apsara* and *D. denisonii* is explained by lineage-specific expansion of tandem repeats rather than by random insertions or sequencing artifacts. Tandem repeat expansion in the mitochondrial control region is a well-documented source of length variation in teleost fishes and is generally associated with the hypervariable nature of this D-loop (Lee et al. 1995; Kundu et al. 2023). Combined with *Dawkinsia filamentosa* (full length 16,598 bp, D-loop 931 bp) in this genus, this situation can be more proved (Sun and Lu 2024). The presence of six repeat copies in *D. denisonii*, contrasted with their absence in *D. apsara*, indicates pronounced interspecific structural divergence and suggests is a more prudent description of the control region within *Dawkinsia*. Such repeat copy number variation may arise through replication slippage and has been reported in several cyprinid lineages, further supporting that the observed pattern represents genuine biological variation, such as *Pethia padamya* (Pan et al. 2023), *Pethia nigrofasciata* (Sun and Lu 2024) and *Pethia stoliczkana* (Qiao et al. 2024), which exhibit mtDNA lengths ranging from 16,792 to 16,966 bp and D-loop regions from 1,134 to 1,340 bp. However, these structural differences can reflect the evolutionary dynamics of lineage specificity, rather than the main criteria for taxonomic classification (Mahai et al. 2024). Therefore, the inference of phylogenetic relationships mainly comes from protein-coding genes, which provide more conserved and comparable evolutionary signals.

The newly sequenced *D. denisonii* mitogenome exhibited 5.24% divergence from the published GenBank sequence, together with 881 SNPs and 22 InDel events, indicating substantial mitochondrial variability within the species. Such levels of divergence are well above the range of technical or assembly-related artifacts and have been documented in several cyprinid taxa as signatures of geographically structured populations or cryptic mitochondrial lineages (Collins et al. 2012). Given that *D. denisonii* is heavily traded in the ornamental fish market, individuals from different river systems may be mixed under the same commercial label, potentially explaining the observed mitochondrial divergence (Jain et al. 2022). Our resequencing effort therefore provides an essential complementary dataset and highlights previously unrecognized intraspecific diversity in *Dawkinsia*.

### Base composition preference and mutation pressure

Both *Dawkinsia* mitochondrial genomes showed significant AT bias (~ 58%), and showed consistent positive AT bias and negative GC bias. This pattern is highly consistent with the report of *Pethia* (Qiao et al. 2024), *Enteromius* (Kundu et al. 2023), *Puntius* (Jang-Liaw et al. 2013), and *Systomus* (Biswal et al. 2017), reflecting the co-evolution trajectory of the mitochondrial genome of Cyprinidae under the action of replication mechanism and chain-specific mutation pressure. This cross-group consistent base composition characteristic strengthens the reliability of the two results of *Dawkinsia*, suggesting that the AT/GC bias can be used as an important background parameter for comparative genomics research of Cyprinidae fish, but its diagnostic value in genus classification is limited.

### Codon usage pattern and selection pressure

Codon usage analysis showed that both species strongly preferred synonymous codons ending with A/U, with leucine (Leu) being the most frequently used, and the use of AUA (encoding methionine) was significantly more than that of the standard codon AUG. This codon usage pattern is consistent with the ‘strong A/U terminal preference’ reported by *Pethia stoliczkana* (Qiao et al. 2024), *Enteromius thysi* (Kundu et al. 2023) and other related species, which is mainly driven by AT mutation pressure at the genome level, rather than translation efficiency optimization. Selection pressure analysis further revealed that all protein-coding genes were subjected to purification selection (Ka/Ks < 1), but there were significant differences among different genes. This differential selection pattern reflects the different functional importance of different genes in the energy metabolism system. Similar gene-specific selection differences have also been reported in studies of *Osteobrama belangeri* (Barman et al. 2017), *Oliotius oligolepis* (Sun et al. 2023) and *Puntius conchonius* (Xu et al. 2015).

### Conservatism of gene overlap/interval

Typical short overlaps such as *ATP8*-*ATP6* and *ND4L*-*ND4* and a small number of unequally spaced regions were observed in both species, which was consistent with the ‘compact’ mitochondrial gene arrangement of *Hampala macrolepidota* (Liu et al. 2015), *P. conchonius* (Xu et al. 2015), *E. thysi* (Kundu et al. 2023), etc., reflecting the long-term selection constraints of carp mitochondria on compact tissue structure. This conservation means that even if the control region length drifts, the relative position of the coding region, the reading frame and the cross-gene boundary relationship remain stable, thus ensuring the robustness of protein synthesis and respiratory chain function.

### Phylogenetic relationship and taxonomic significance

ML and BI analyses based on the mitochondrial PCGs, rRNA genes and tRNA genes showed that *D. denisonii* first formed a strongly supported sister relationship with *D. chalakkudiensis*, which together clustered with *D. tambraparniei*; this clade was subsequently grouped with *D. apsara*. All *Dawkinsia* species formed a well-supported monophyletic group. In this study, only the mitochondrial D-loop were not used for phylogenetic reconstruction because they allow reliable codon-based alignments and retain sufficient phylogenetic signal at the interspecific level. In contrast, the D-loop were excluded due to their high sequence variability, potential substitution saturation, and alignment ambiguities caused by length variation and tandem repeats. This is consistent with the results of recent studies on multiple mitochondrial systems: in the phylogenetic tree of *P. stoliczkana*, *P. ticto*, *P. padamya*, and *D. denisonii* were clustered into adjacent branches (Qiao et al. 2024); *Oliotius oligolepis* is also located within Smiliogastrinae, adjacent to the *Puntius* group (Sun et al. 2023). Previous phylogenetic studies of *Dawkinsia* and its related genera mainly relied on mitochondrial short fragments, such as 16S rRNA and *Cytb*, which generated inconsistent or weakly supported topologies in multiple interspecific relationships (Pethiyagoda et al. 2012). In contrast, the phylogenetic framework inferred from complete mitochondrial protein-coding genes in this study provides a more robust and well-supported topology that addresses these previously ambiguous nodes through a consistent stepwise clustering pattern within *Dawkinsia*. The strong support values recovered at the main node indicate that the use of complete mitochondrial genomes greatly improves the resolution of phylogenetics and clarifies previously uncertain interspecific relationships, thereby strengthening the systematic position of species within the genus.

In the past, *Dawkinsia* was seen as a composite subgroup of *Puntius* (Zheng et al. 2016; Tan and Armbruster 2018). However, with the accumulation of high-throughput sequencing data, many studies have pointed out that *Puntius* is a multi-lineage group, while *Dawkinsia*, *Pethia*, and *Sahyadria* are independent evolutionary branches. The phylogenetic tree showed that some species traditionally classified as *Puntius* were not clustered in the core *Puntius* branch, but formed closer phylogenetic relationships with *Dawkinsia* and *Pethia*, respectively. This result provides more solid molecular evidence for the taxonomic revision of *Dawkinsia* separated from *Puntius*, and also suggests that the traditional concept of *Puntius* may still contain heterogeneous groups that need to be further clarified. Recent studies on the genus *Enteromius* in Africa have also revealed significant phylogenetic differentiation, indicating that the phylogenetic relationship of small carps is complex and often accompanied by geographical isolation (Kundu et al. 2023).

## Conclusions

In this study, the complete mitochondrial genome sequence of *D. apsara* was obtained for the first time, and *D. denisonii* was re-sequenced, which significantly enriched the genome resources of the genus *Dawkinsia*. By combining this sequence with the extended Smiliogastrinae mitochondrial genome dataset, we verified and refined the phylogenetic relationship between *Dawkinsia* and its related genera such as *Puntius* and *Pethia* at the genome scale. In addition, the comparative analysis further revealed significant differences in the structure of the mitochondrial control region between the two species, indicating that there is an evolutionary differentiation at the mitochondrial genome level within the *Dawkinsia* genus. Taken together, this study not only deepens the understanding of the phylogenetic relationship and evolutionary differentiation pattern of the genus *Dawkinsia* at the complete mitochondrial genome level, but also provides new molecular evidence for the taxonomic study of the genus *Dawkinsia* and its related groups.

## Additional information

### Use of AI

No use of AI was reported.

### Author ORCIDs

Yang Xu https://orcid.org/0009-0005-4440-2110

Cheng-He Sun https://orcid.org/0000-0002-0650-5443

Chang-Hu Lu https://orcid.org/0000-0002-8607-7134

### Data availability

All of the data that support the findings of this study are available in the main text.
